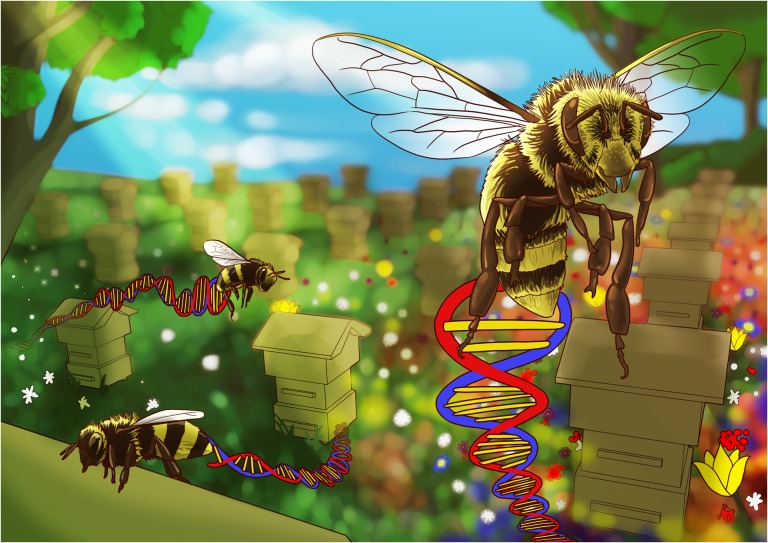# Bee healthy! Honeybee physiology reflects landscape and supports conservation

**DOI:** 10.1093/conphys/cox024

**Published:** 2017-04-24

**Authors:** Björn Illing

**Affiliations:** 1 ARC Centre of Excellence for Coral Reef Studies, James Cook University, Townsville, QLD 4811,Australia

There are many threats to honeybees, but new findings from Alaux *et al.* (2017) show that the bees’ physiology can help detect the best-suited landscapes for the diligent pollen collectors and aid in their conservation.

Up to 10% of global food production—including many of our favourite foods, such as apples and almonds—relies on honeybees. However, increased temperatures associated with climate change can lead to a mismatched calendar for when plants begin flowering and when bees come out of hibernation. In the same way, pesticides, parasites and habitat loss can also affect bee survival. Amongst these stressors, fragmented and deteriorated landscapes have strongly contributed to the pressure on honeybee health and their ability to survive winter months.

Typically, bee population health is assessed by distribution patterns and overall numbers. But when landscapes are seriously altered, the deleterious effects on bee populations can only be detected once the population has already started to decline!

So, how can a stressful landscape be detected earlier so that bee populations can be protected before it is too late? Alaux *et al.* (2017) designed physiological experiments to find out what environments benefit bee health the most. The team exposed different colonies of honeybees to different amounts of either honey-yielding catch crops (grown between successions of main crops) or semi-natural habitats and took physiological measurements between autumn and spring. They measured expression levels of a gene responsible for bee longevity and nutrient storage as well as the overall body fat of the bees. Alaux’s team also looked at other metrics of bee health, such as parasitic mite infestation, reproductive state, and the amount and composition of pollen the bees gathered from the various landscapes. The team put all of these numbers into a model to determine which factor contributed most to the ability of the bees to survive winter.

Alaux *et al.*(2017) demonstrated that measuring the physiology of a few bees is a good way to determine the whole population’s health. Using their model, the team observed the level of mite infestation and the physiological parameters to help best predict the overwinter survival of honeybee colonies. For the first time, they could link bee physiology with landscape patterns, specifically suggesting that bee health is better under semi-natural habitats as opposed to landscapes that are enriched with catch crops.

What, then, is the solution to best protect bee populations? Alaux and team recommend that managers add artificial bee pastures with a high diversity of flowering plants to aid in protecting the semi-natural habitat that bees prefer—the habitat that helps them stay the healthiest.

This approach is promising. It shows that physiological tools can assist in better understanding the cause-and-effect mechanisms that can help with timely decision-making and more sustainable conservation management and policy. Another very practical benefit is that we might be able to keep apples, almonds, and—of course—honey, on our daily menus.

Illustration by Erin Walsh; Email: ewalsh.sci@gmail.com

**Figure:**